# Adolescents and Young Adults With Acute Lymphoblastic Leukemia: Qualitative Barriers and Facilitators to Guideline-Concordant Care

**DOI:** 10.1177/10732748261469238

**Published:** 2026-07-20

**Authors:** Allison C. Grimes, Julie A. Wolfson, Charlotte L. Kerber, Kristin M. Bingen, Melissa P. Beauchemin, Koh B. Boayue, Jennifer M. Levine, Michele A. Scialla, Wendy L. Woods, Jane J. Liu, Olivia Ponce, Sarah L. Vargas, Anjali S. Advani, George J. Chang, Emily K. Curran, Dawn L. Hershman, Selina M. Luger, Kristen M. O’Dwyer, Wendy Stock, David S. Dickens, Michael E. Roth, David R. Freyer, Brad H. Pollock, Lillian Sung, Susan K. Parsons

**Affiliations:** 1 Division of Pediatric Hematology-Oncology, 222550 University of Texas Health Science Center San Antonio, San Antonio, TX, USA; 2 Division of Pediatric Hematology-Oncology, 9968 University of Alabama at Birmingham, Birmingham, AL, USA; 3 Institute for Cancer Outcomes and Survivorship, 9968 University of Alabama at Birmingham, Birmingham, AL, USA; 4 Department of Public Health Sciences, 240462 University of California, Davis, Davis, CA, USA; 5 Department of Pediatrics, 547971 Medical College of Wisconsin, Milwaukee, WI, USA; 6 School of Nursing, 15760 Columbia University Irving Medical Center, New York, NY, USA; 7 Division of Pediatric Hematology-Oncology, 130373 University of New Mexico Cancer Center, Albuquerque, NM, USA; 8 Division of Pediatric Hematology-Oncology, Children’s National Medical Center, Washington, DC, USA; 9 Division of Pediatric Hematology-Oncology, 547296 Nemours, Wilmington, DE, USA; 10 Division of Pediatric Hematology-Oncology, 23195 Blank Children’s Hospital, Des Moines, IA, USA; 11 6497 OSF Healthcare System, Peoria, IL, USA; 12 Division of Hematology-Oncology, Illinois CancerCare, Peoria, IL, USA; 13 151218 Children’s Oncology Group, Monrovia, CA, USA; 14 Department of Hematology and Medical Oncology, 2569 Cleveland Clinic, Taussig Cancer Institute, Cleveland, OH, USA; 15 Department of Colon and Rectal Surgery and Department of Health Services Research, 4002 The University of Texas, MD Anderson Cancer Center, Houston, TX, USA; 16 Division of Hematology-Oncology, 199712 University of Cincinnati, Cincinnati, OH, USA; 17 Division of Hematology-Oncology, 640917 Columbia University, New York, NY, USA; 18 Division of Hematology-Oncology, 312087 University of Pennsylvania, Philadelphia, PA, USA; 19 Division of Hematology-Oncology, 578154 University of Rochester, Wilmot Cancer Institute, Rochester, NY, USA; 20 Division of Hematology-Oncology, 21727 University of Chicago Medicine, Chicago, IL, USA; 21 Division of Pediatric Hematology-Oncology, 21782 University of Iowa, Iowa City, IA, USA; 22 Division of Pediatric Hematology-Oncology, MD Anderson Cancer Center, Houston, TX, USA; 23 Cancer and Blood Disease Institute, 337887 Children’s Hospital Los Angeles, Los Angeles, CA, USA; 24 Division of Pediatric Hematology-Oncology, 7979 The Hospital for Sick Children, Toronto, ON, USA; 25 Division of Hematology, Oncology and Institute for Clinical Research and Health Policy Studies, 550030 Tufts Medical Center, Boston, MA, USA

**Keywords:** adolescent and young adult (AYA), acute lymphoblastic leukemia (ALL), guideline concordant care, treatment guidelines, pediatric-inspired therapy

## Abstract

**Introduction:**

Progress in acute lymphoblastic leukemia (ALL) affecting adolescents and young adults (AYAs), aged 15-39 years, has been challenged by aggressive disease biology, low clinical trial participation, unique supportive care needs, and heterogeneity across treatment settings with lack of age-based standardized therapy. In 2012, the National Comprehensive Cancer Network (NCCN) first issued AYA ALL treatment guidelines. In the absence of formal evaluation of the 2012 NCCN guidelines on the delivery of guideline-concordant care at National Cancer Institute (NCI) Community Oncology Research Program (NCORP) practices, we conducted a qualitative study, as part of the Children’s Oncology Group-led cancer care delivery trial ACCL16N1CD.

**Methods:**

Structured focus groups, moderated by study members, were convened to identify barriers and facilitators to NCCN guideline-concordant treatment *delivery* and *documentation*. Healthcare professionals at NCORP sites that activated ACCL16N1CD were invited to participate if they were involved in AYA ALL care. Nine focus groups were held with 55 participants. Nominal group technique was used to rank statements about delivery and documentation. Qualitative data were analyzed using directed content analysis methodology to describe facilitators and barriers and assess emerging themes.

**Results:**

Five main themes were identified for delivery (Care Model, Care Organization, Supportive Care, Therapeutic Approach, Individual Factors [patient; provider]) and six for documentation (Care Model, Hospital Type, Care Organization, Supportive Care, Therapeutic Approach, Individual Factors) of NCCN-concordant treatment. Supportive Care was ranked first for delivery of guideline-concordant care and Care Organization was ranked first for documentation, with Individual Factors ranking close behind for both. Theme ranking varied by focus group type and professional roles of participants.

**Conclusion:**

Identified barriers and facilitators impacting guideline-concordant care of AYA ALL across NCORPs were aligned with institutional supportive care resources, care organization for AYA, and individual factors. Top ranked statements may be utilized as implementation strategies in future care delivery trials.

## Plain Language Summary

In recent decades, adolescent and young adults (AYA) with acute leukemia have not experienced the same gains in treatment tolerance and survival compared to younger children and older adults. Specific guidelines have been developed for AYA acute leukemia treatment, yet many of these patients do not receive guideline-based therapy, particularly in community settings where most receive cancer care. This study includes a series of focus groups, held to discuss directly with community healthcare professionals who care for AYA with leukemia, what specific challenges and aids influence whether or not AYA with leukemia are able to receive guideline-based therapy in their settings. From these discussions, key factors that influence the ability to deliver therapy that is guideline-based include having supportive care resources in place for these patients, having or lacking organization of the care, and some challenges at the level of the patient or provider that may influence the care that is delivered.

## 1. Introduction

Survival among individuals diagnosed with acute lymphoblastic leukemia (ALL) between age 15 to 39 years (AYAs: adolescents and young adults) lags behind those diagnosed as children.^1-7^ Differential outcomes between AYAs and children with ALL are partly attributed to aggressive disease biology and differences in management.^8^ Most AYAs with ALL are treated in community settings in increasing proportions as they age.^9-11^ Thus, it is essential to characterize delivery of cancer care in addition to clinical and biological aspects of AYA ALL.

As AYA ALL survival is associated with clinical trial enrollment and treatment approach,^12^ the National Cancer Institute (NCI) has prioritized work in AYA cancer and clinical trial enrollment; nevertheless, AYAs remain under-represented.^13-15^ To address ALL outcome disparities, the National Comprehensive Cancer Network (NCCN) established clinical practice guidelines for AYA in 2012. Given superior AYA ALL outcomes with pediatric rather than adult regimens,^16-21^ NCCN incorporated age-specific treatment into ALL guidelines (e.g., AYA [15-39 years] vs. adults ≥40 years).^22^ AYA ALL guidelines remained consistent from 2012 to 2016,^22,23^ recommending pediatric-inspired therapy or clinical trial enrollment for AYA with Philadelphia chromosome negative (Ph-neg) ALL (T-cell or B-cell). For Philadelphia chromosome positive (Ph-pos) ALL, guidelines recommended multi-agent chemotherapy with a tyrosine kinase inhibitor.

**Figure 1.**
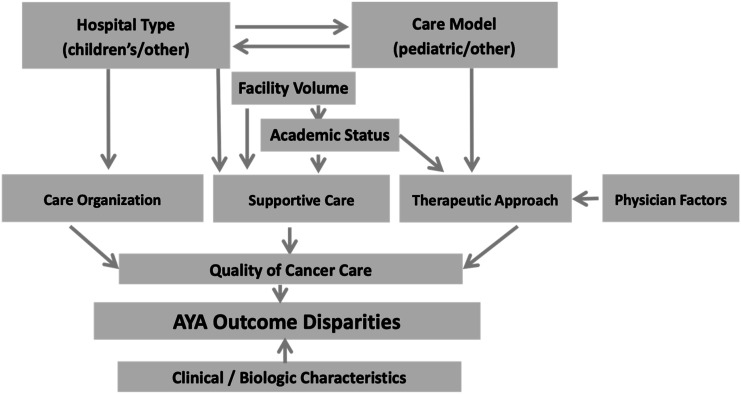
Adolescent young adult (AYA) cancer care delivery model (Wolfson J and Bhatia S)

Given most AYA with ALL are treated in their community, the NCI Community Oncology Research Program (NCORP) is the ideal setting to examine care delivery for this population. The NCORP network includes stand-alone children’s hospitals, adult-only hospitals, and general hospitals with embedded pediatrics (i.e., mixed) across the United States (US), aiming to improve cancer research and trial access for patients closer to home.^24-28^ Understanding that evidence-based system-, provider- and patient-level factors contribute to AYA cancer disparities (Figure 1),^1-15,29^ the ACCL16N1CD study examined guideline-concordant care (GCC; specifically, NCCN guidelines) among AYA with ALL across the NCORP. Characteristics of healthcare settings where AYA with ALL receive care have been described.^30^ Here we present qualitative study findings evaluating the ACCL16N1CD objective to identify for AYAs with ALL, targetable structure- and process-level barriers and facilitators that will increase the proportion of patients having a documented treatment plan and receiving treatment according to NCCN guidelines.

## 2. Methods

ACCL16N1CD (NCT03204916) was a retrospective, multi-center intergroup study for the NCI Cancer Care Delivery Research (CCDR) program implemented at NCORPs^27,28,31^ and led by the Children’s Oncology Group (COG) in collaboration with adult-focused NCORP Research Bases (Alliance for Clinical Trials in Oncology [Alliance], ECOG-ACRIN Cancer Research Group [ECOG-ACRIN], SWOG Cancer Network [SWOG]).

### 2.1. Institutional Participation

Institutional eligibility for ACCL16N1CD included (a) willingness to participate in all study aims, (b) classification as an NCORP between December 2017 and April 2022, and (c) treatment of ≥1 patient 15-39 years of age with B- or T-cell ALL between 2012 and 2016, regardless of clinical trial participation.

### 2.2. Focus Group Recruitment

Healthcare professionals at participating NCORPs caring for AYA ALL were eligible for focus groups (FGs), including (but not limited to) physicians, nurses, social workers, pharmacists, and clinical research associates; trainees were ineligible. NCORP leadership and/or research staff distributed standardized electronic invitations to eligible individuals (Appendix I); interested volunteers completed an electronic questionnaire providing role/training details regarding the location at which they delivered care (clinical facility [CF]: study-defined entity^30^; Appendix II). The study team selected FG participants from the volunteer pool for representativeness by CF model (pediatric-only, adult-only, or mixed), academic status, NCORP classification (community vs. minority-underserved),^26^ geography (US region and rural vs. urban), oncology specialty (pediatric vs. adult), and professional role. Separate FGs were conducted with physicians and non-physicians to avoid potential response censoring and bias based on hierarchical relationships, plus mixed physician/non-physician FGs to elicit new ideas. More than one individual from each NCORP could participate. Volunteer selection occurred selectively in a rolling fashion as focus groups sessions were filled. For example, for non-physician focus groups, volunteers available for a specific session were selected to account for variability across facility model first, followed by professional role, and then the remaining factors as listed above (academic status, NCORP classification, etc.).

### 2.3. Data Collection

FGs of 5-8 participants lasting 1.5-4 hours each were held either in person or virtually (due to COVID-19 travel limitations); in-person FGs were held, where possible, adjacent to professional meetings, and virtual FGs used Zoom video conferencing. FG sessions included volunteer participants, research team members, and COG operations staff. FGs were recorded and professionally transcribed, with thematic notes following the moderator guide (Appendix III) taken to augment the analysis; transcripts were not returned to participants. All data collection and analyses were conducted by study team members who received the same methodological training, including focus group facilitator training with the Public Health Institute’s Survey Research Group (ACG, CLK, KMB, JAW, MPB, KBB, JML, MS, WLW). FG research team members were female and included oncology clinician scientists and research nurses who did not have a prior relationship established with volunteer participants.

Nominal group technique (NGT) was used as a structured discussion method, enabling inclusive group brainstorming and prioritization of ideas for consensus that limits potential dominating perspectives.^32,33^ Per NGT procedures, the group first reviewed a brief background on NCCN guidelines for AYA ALL and the FG questions, specifically: (a) barriers to delivering GCC, (b) facilitators to delivering GCC, (c) barriers to documenting GCC, and (d) facilitators to documenting GCC. Participants next generated ideas independently, then presented in turn to the group at large until no new ideas were generated. Ideas were discussed for clarification and recorded by the facilitator. Participants then rank-voted all ideas twice, before and after a group discussion. In-person participants had 15 votes per round that could be allocated across ideas in any quantity to represent a weighted rank. For virtual FGs, ranking was done electronically using the Poll Everywhere® platform. The final ranked statements resulted from the sum of second-round votes received in-person or the actual ranking on the electronic platform. This process was repeated for each FG question. FGs were conducted until thematic saturation was achieved with no new themes emerging.

### 2.4. Analysis

Qualitative data, consisting of ranked statements generated by participants describing barriers and facilitators to GCC, were analyzed using directed content analysis with an assessment of emerging themes (Dedoose Version *9.2.012* [Computer Software]. Los Angeles, CA: SocioCultural Research Consultants, LLC. www.dedoose.com.).^34^ A codebook was developed based on structure- and process-level categories from the conceptual model of AYA cancer care disparities (Figure 1; Appendix IV).^29^ Seeking thematic commonality, emerging concepts that did not fall within these categories were added. Two study team members conducted independent coding (ACG, CLK) and reviewed results with two additional study team members (JAW, KMB) for consensus. The reporting of this study conforms to the COREQ guidelines. The study was approved by the NCI Pediatric Central Institutional Review Board (IRB) on September 1, 2017. Documentation of consent requirement was waived given the nature of the study. This study was conducted in accordance with the Helsinki Declaration of 1975, as revised in 2024.

## 3. Results

### 3.1. Participants

Among 167 volunteers, n=158 were eligible and n=55 were selected and participated in nine FGs (Table 1). Most participants were female (80%) and physicians (53%). Nurses represented 46% of non-physicians and 22% of all participants. Participants represented 31 CFs (Appendix VI) spanning adult (15%), pediatric (31%), and mixed (54%) CFs, with more from community practices (58%) than academic institutions (31%) or NCI-designated Comprehensive Cancer Centers (9%). Few (11%) worked in rural settings. (Details provided in Appendix VII).

**Table 1.** Characteristics of Focus Group Participants: Self-Report

​	Total	^§^Mixed	^§^Non-physician	^§^Physician
	*N*=55 (%)	Groups 1 and 3 *n*=14 (%)	Groups 2 and 4 *n*=18 (%)	Groups 5 and 6 *n*=23 (%)
Sex				
*Male*	11 (20)	3 (21)	0 (0)	8 (35)
*Female*	44 (80)	11 (79)	18 (100)	15(65)
Profession				
Physician	29 (53)	6 (43)	0 (0)	23 (100)
Non-Physician	26 (47)	8 (57)	18 (100)	0 (0)
*Registered Nurse*	12	5	7	--
*Social Worker*	4	2	2	--
*Psychologist*	1	0	1	--
*Pharmacist*	1	0	1	--
*Nurse Practitioner*	2	0	2	--
*Clinical Research Associate*	5	1	4	--
*Other*	1	0	1	--
Institution Type				
*Adult*	8 (15)	1 (7)	5 (28)	2 (9)
*Pediatric*	17 (31)	5 (36)	5 (28)	7 (30)
*Mixed*	30 (54)	8 (57)	8 (44)	14 (61)
Practice Setting				
Academic program	17 (31)	3 (21)	8 (44)	6 (26)
Community oncology program	32 (58)	9 (65)	7 (39)	16 (70)
NCI-designated comprehensive cancer center	5 (9)	2 (14)	2 (11)	1 (4)
Unknown	1 (2)	0 (0)	1 (6)	0 (0)
Practice Location				
Urban	49 (89)	13 (93)	17 (94)	19 (83)
Rural	6 (11)	1 (7)	1 (6)	4 (17)
Number of AYA Treated per Year				
<5	5 (9)	1 (7)	2 (11)	2 (9)
5-10	13 (24)	3 (21)	1 (6)	9 (39)
10-25	22 (40)	6 (43)	10 (56)	6 (26)
>25	13 (24)	4 (29)	3 (17)	6 (26)
Unknown	2 (4)	0 (0)	2 (11)	0 (0)
Number of ALL Treated per Year				
<5	9 (16)	4 (29)	2 (11)	3 (13)
5-10	9 (16)	1 (7)	3 (17)	5 (22)
10-25	17 (31)	4 (29)	4 (22)	9 (39)
>25	18 (33)	5 (36)	7 (39)	6 (26)
Unknown	2 (4)	0 (0)	2 (11)	0 (0)

^§^Focus groups were comprised of either (a) only physicians, (b) only non-physicians, or (c) both physicians and non-physicians (mixed). Abbreviations: National Cancer Institute (NCI), Adolescent and Young Adult (AYA), Acute Lymphoblastic Leukemia (ALL).

### 3.2. Themes

**Figure 2.**
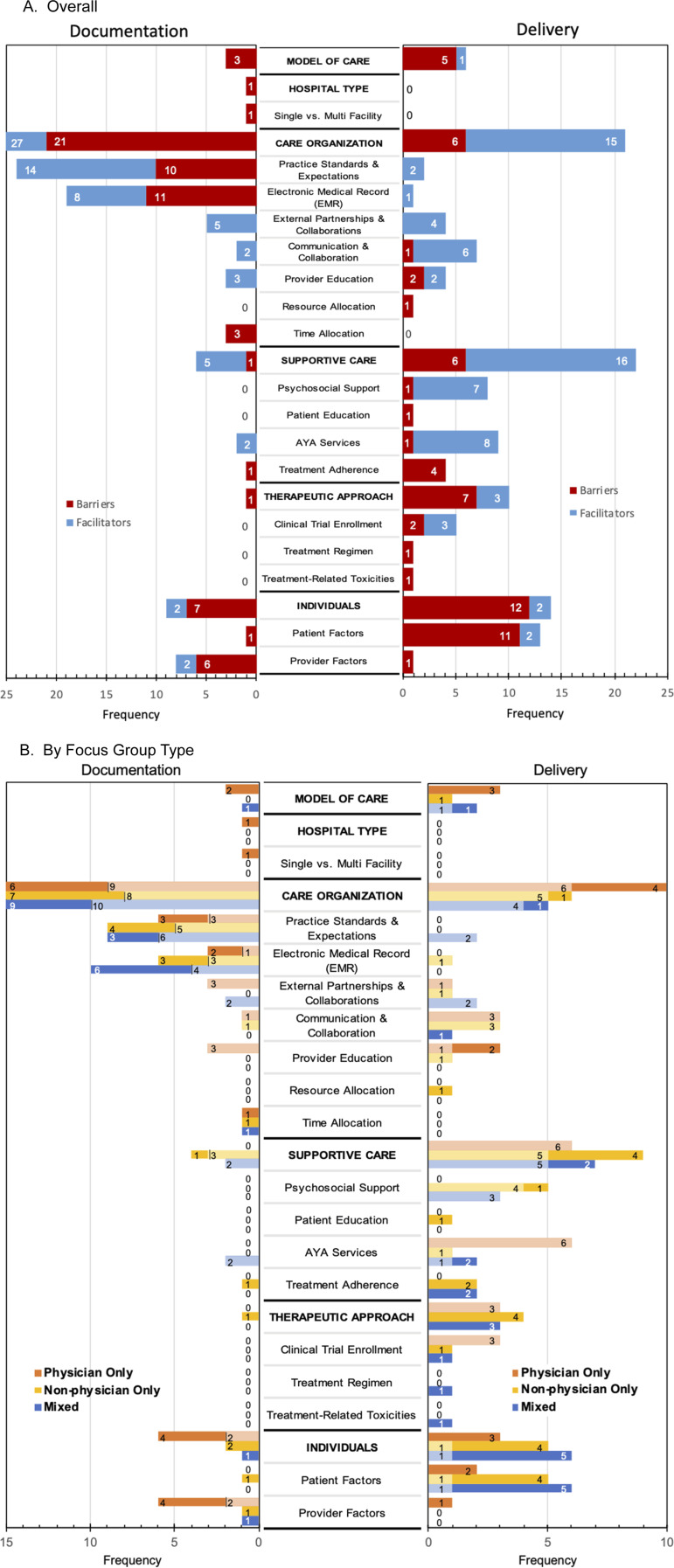
Coding frequency of themes by focus group type and identification as either a barrier or facilitator for documentation and delivery of NCCN-concordant care for AYAs with ALL. Main themes (all caps) and sub-themes (denoted under corresponding main theme) were generated by all focus groups throughout study period. Facilitators are displayed in lighter shade and numbered closest to *y*-axis; barriers are in darker shade and numbered furthest from *y*-axis. All statements coded by sub-theme are also coded by corresponding main theme; however, not all statements coded by main theme are also coded for by sub-theme

The highest ranked statements from each FG are presented in Table 2. Frequencies of coded themes and sub-themes are illustrated in Figure 2; for each theme, facilitator and barrier statements were combined given their overlapping nature. For example, one group posed leadership support as a facilitator, while another group posed lack of leadership support as a barrier; both statements are coded under the same theme. A comprehensive table of coded ranked statements is in Appendix V.

**Table 2.** Top Five Ranked Statements by Year and Focus Group Type

Rank	A. Barriers to delivery					
	2019			2021		
	Group 1 Mixed	Group 2 Non-physician only	Group 3 Mixed	Group 4 Non-physician Only	Group 5 Physician only	Group 6 Physician Only
1	Inability to give subsequent therapies due to treatment toxicity/AEs of drugs/infection.	Coordinating treatment with patient schedules as AYAs have work, families, and are less flexible.	Poor adherence to medications/appointments.	Compliance of this age group (taking medications, reporting side effects, waiting until very sick before asking for help).	Limitation/knowledge gap in local community-based providers leads to reduced cancer center referrals for ALL; provider-dependent.	Complexity of care for patient population presents challenges in getting patients easily in and out of clinic.
2	AYA referral process/source (adult PCP to adult oncology vs. pediatric PCP to pediatric oncology); inconsistent placement of patient.	Concerns about patient compliance.	AYAs are underinsured or have no insurance, also lower socioeconomic status.	Financial issues and lack of support interferes with care, ability to take time off, transportation, etc.	Age restrictions: younger AYA treated at pediatric site vs. older AYA treated at adult site with varying care/access/assessment.	Insurance status dictates location of care in some cases and drug coverage.
3	Increased adherence issues in AYA (e.g., medications).	Complexity of protocols/publications without adequate guidance.	Low clinical trial access/availability for AYA (especially in COG or non-COG sites).	Poor health literacy impacts information provided explaining treatment, understandable language, fertility issues in this age group.	Training deficiencies in pediatric-inspired regimens for adult oncology related to rarity of disease/reduced exposure to ALL leads to gap in knowledge (need for workshop, CE).	Institutional restrictions on age limitations that can be seen at certain locations.
4	Social and cultural issues impact treatment decisions (including geographic/travel challenge).	Higher cumulative doses in pediatric protocol, concern for increased toxicities.	Poor communication between pediatric and adult providers and community providers.	Determination of where a particular AYA will “fit in” for treatment (pediatric vs. adult).	Socioeconomic discrepancies.	Some subspecialists won’t see patients over age 18 at stand-alone children’s hospital.
5	Complexity of AYA pediatric backbone protocols.	Institutional resources, more is needed for pediatric regimens.	Transition to age of majority on therapy leads to challenges with buy-in/consent/adherence.	Gap in research studies available for AYA population, may not be eligible for clinical trial based on age.	Challenges in obtaining preferred laboratory/molecular sequencing assessments (including requiring case-by-case authorization).	Social workers, navigators and support staff may be less familiar with AYA specific needs/resources.

Rank	B. Facilitators to delivery					
	2019			2021		
	Group 1 Mixed	Group 2 Non-physician only	Group 3 Mixed	Group 4 Non-physician only	Group 5 Physician only	Group 6 Physician only
1	Oncology team approach to AYA care including navigator/coordinator (including patient follow-up, phone calls, education, communication established).	Open-sharing of roadmaps from protocols (e.g., COG) chemotherapy, labs, etc.	Standardized order sets (treatment plan) including chemotherapy, labs, supportive care (guideline-based).	Transparency with patients, therapy schedule reminders, how to prepare for visits, personalized medication calendars.	AYA focused research.	Educating providers so that they are more confident/comfortable with AYA care like additional AYA-specific support staff (i.e. AYA social worker).
2	Psychosocial support (travel, insurance, meals, bills, etc.).	Regularly scheduled team meetings for communication.	Dedicated oncology social worker.	Rapport with and accessibility of the medical team, including psychosocial.	Front-line NPs/APNs and CRAs, having an AYA champion, RNs assigned to disease team (i.e. leukemia).	Existing collaboration with adult and pediatric oncology including familiarity with NCCN guidelines and active studies available across pediatric/adult practices.
3	Parent or caregiver involvement.	Multi-disciplinary team helps to provide all types of needed care.	Sharing templated/standardized processes across institutions.	Communication between doctors and research staff across pediatric and adult units, enhanced by EMR, easy to access records.	Collaboration between pediatric and adult teams; having someone embedded in the program that understands AYA medicine and participates in tumor boards (local/external).	Medical oncologist(s) with COG membership allows for adult oncology to see/enroll patients onto COG studies and access to roadmaps for off-study patients.
4	AYA best practice care guideline for all institutions to follow.	Building relationships with AYA - counselor especially for AYA.	High capacity/engaged pharmacist.	Parental support (the more involved the parents, the more compliant the AYA).	Advocacy from leadership (i.e. cancer center leader), prioritizing AYA.	Quality of the NCCN guidelines, ease for users, could be made better with link/access to roadmaps for simplicity.
5	Having an institutional guideline in place for which AYAs go to pediatric vs. adult oncology.	Hands-on training at pediatric institution/resources and networking.	Philanthropy (to support staff, programming), access to local housing/support for out-of-town AYA.	Accessibility to resources (financial, insurance).	Sharing eligibility information/screening to cross enroll AYAs onto clinical trials.	Providers advocating for AYA issues at the institutional level (e.g., insurance, expanding age seen by sub-specialists, more access to other sub-specialists).

Rank	C. Barriers to documentation					
	2019		2021		2022	
	Group 1 Mixed	Group 2 Non-physician only	Group 3 Mixed	Group 4 Non-physician only	Group 5 Physician only	Group 6 Physician only
1	Multiple EMR systems across spectrum of care sites; lack of access by staff (CRA, RN, etc.) that don’t communicate; electronic and paper duplicity.	Different documentation practices by setting (in/outpatient).	Paper + EMR combined systems lead to duplication of effort, poor efficiency, and inconsistencies; challenges with varied communication/documentation of home infusions.	EMR documentation difficulty: lack of electronic roadmap, challenge reviewing treatment plan, transfer plan to roadmap, lack of information via EMR for open/eligible trials, hard to track treatment changes, lack of interface with in/outpatient.	Lack of awareness of disease-specific NCCN guidelines for AYA (i.e. ALL).	Lack of awareness that specific NCCN guidelines exist; lack of trigger in ALL population to consult NCCN guidelines.
2	Knowing what are elements that need to be documented; variability of what is documented.	Lack of electronic roadmap for treatment; documentation is not always up to date.	Information in EMR difficult to find/access; completing EMR is time-consuming; there are multiple EMR locations to document AE’s.	Provider doesn’t update information in EMR or roadmap (or days entered incorrectly).	Lack of utilization or resistance of NCCN guidelines.	No specific practice standard (formal template or included in consent) for documenting NCCN therapy, but rather each cycle/phase of therapy planned/given.
3	Multiple areas to document same info in EMR leading to inconsistent data capture; too many places to look for where and/or what to document.	Paper vs. electronic documentation in EMR.	Medication reconciliation is cumbersome and not user-friendly.	Lack of patient education (including oral medications).	Lack of training around NCCN guidelines, especially in pediatric-trainees where less guidelines exist that are pediatric-specific.	Multiple practice locations and providers (including treatment off-site) with differing health record systems and familiarity with NCCN guidelines for AYA.
4	Lack of consistent provider documentation; no way to search EMR due to variability.	Time.	Varied consent/documentation processes and roles in pediatric vs. adult oncology; increased patient volume in adult oncology is RVU-driven.	No roadmap or only hard copy exists.	Lack of familiarity with required documentation: differences in patients on/off-study; whether NCCN guidelines are included.	Documentation of NCCN-concordance in notes less prevalent practice in pediatric oncology (especially in ALL).
5	Time; having adequate time to document.	Hard to find all the information.	High workload and high patient-to-staff ratios.	Long treatment, reluctance of patients to keep timeline of therapy (leads to treatment delays).	Documentation is time-consuming and difficult to be complete in a busy practice; no existing templates for guideline.	Multiple locations in the health record to document therapy and unclear who should be responsible for documenting various aspects of therapy.

Rank	D. Facilitators to documentation					
	2019		2021		2022	
	Group 1 Mixed	Group 2 Non-physician only	Group 3 Mixed	Group 4 Non-physician only	Group 5 Physician only	Group 6 Physician only
1	Knowing what to document and who documents (standard for documentation); specified button in Beacon.	Standardized approach for AE; does modifications in chart.	Oncology summary/single location for patient information of diagnosis and treatment.	Multi-disciplinary team meetings/discussions/huddles for planned care (treatment, scans, etc.); staff with access to both inpatient and outpatient EMR.	Standardized implementation of NCCN guidelines in COG protocols.	Awareness that the guidelines exist; education/awareness of providers/staff.
2	Note templates, dot phrases in EMR.	Having an EMR.	Cooperative groups to work with industry for oncology-specific EMR/universal EMR.	Oral medication calendars for patient each visit (assists with patient education, clarity of medications); patient and parent support; medication reconciliation with patient/family (including providing copy of roadmap to patient); referrals to support services for poor adherence.	Including guidelines in the “How I Treat” and/or ASH publication series.	Practice changes around documentation that includes staff/provider training for this to be standard practice.
3	Single location for treatment plan documentation.	Treatment plan within EMR (Beacon).	AYA working group to create checklist of documentation needs on/off trial (build templates, hard-stops, dot phrases, exportable AYA module).	Development of roadmaps at site (paper) that reflect treatment protocol; roadmap follows patient from inpatient to outpatient.	Consensus among providers around NCCN concordant treatment.	Auto-text or dot phrases; add a link in the protocol or add a check box in the consent document to use that mentions “NCCN guideline-based care”.
4	“AYA Navigator” as designated point person responsible for documenting information; creates and consistently uses template.	Roadmap that outlines treatment, labs, etc.	Share best practices with other institutions (i.e. AYA RI network).	Presence of a patient navigator.	Additional education for providers and trainees to improve awareness.	Formal discussion of patient (journal club, tumor board, group review).
5	Utilization of consent for all patients not only study patients.	One roadmap that “follows” patient.	Clinical nurse educator; audits.	SOP to aid with standardized patient care at institution (supportive care, hydration).	Inclusion of NCCN hyperlinks from COG content.	Strong CRA or regulatory/compliance staff; ensures adequate documentation.

Statements were made by focus group participants then clarified and edited using the nominal group technique approach. Abbreviations: Electronic Medical Record (EMR), Clinical Research Assistant (CRA), Registered Nurse (RN), National Comprehensive Cancer Network (NCCN) Adolescent and Young Adult (AYA), Acute Lymphoblastic Leukemia (ALL), Adverse Event (AE), Relative Value Unit (RVU), Children’s Oncology Group (COG), American Society of Hematology (ASH), Responsible Investigator (RI), Standard Operating Procedure (SOP), Primary Care Provider (PCP), Continuing Education (CE), Nurse Practitioner (NP), Advanced Practice Nurse (APN).

#### 3.2.1. Delivery of GCC

Five main ***themes*** with 15 associated *sub-themes* arose for delivery of GCC, with ***Supportive Care*** and ***Care Organization*** most frequent, followed by ***Individuals*** (*Provider Factors* and *Patient Factors* capturing specific behaviors or beliefs). Healthcare professionals across FG types identified ***Supportive Care*** (e.g., psychosocial factors) as the primary influence for AYA ALL care delivery, mostly as facilitators for *AYA Services* and *Psychosocial Support*. ***Care Organization*** ranked similarly, with strong sub-themes of *Communication & Collaboration*, *External Partnerships & Collaborations*, and *Provider Education.* ***Individuals*** ranked third, mostly due to *Patient Factors* like adherence presenting barriers to GCC delivery. The remaining two of the top five delivery themes included ***Care Model*** and ***Therapeutic Approach***.

Prominent sub-themes within ***Supportive Care*** included *Psychosocial Support* and *AYA Services*. Presence of *Psychosocial Support* (reflected in top-ranked statements as assistance scheduling appointments and navigating care-related finances) was emphasized as a facilitator to initiating and completing GCC by non-physicians and physicians: *“… reminding them of their therapy schedule, inpatient or outpatient, what to expect, what to bring, and timeframes”* [Facilitator, Non-Physician, 2021]. Absence of this support is a noted barrier to GCC: *“I don’t have all the [support] resources here that an AYA patient would need”* [Barrier, Physician, 2021].

Across FG types, *AYA Services* facilitated GCC. Participants agreed in top-ranked statements that AYA-dedicated staff and resources, provider advocacy, and AYA-focused research improved care: *“I think what would help would be, some type of [AYA] coordinator and navigator… it’s having a team approach. There’s a lot of different parts. And having everyone work together… helps when you all know you’re working towards something”* [Facilitator, Mixed, 2019].

The ***Care Organization*** sub-theme *External Partnerships & Collaborations* was identified primarily as a facilitator across FG types, highlighting potential implementation strategies like sharing templated/standardized processes across institutions, embedded link/access to roadmaps within NCCN guidelines, open-sharing of roadmaps from COG protocols, and having AYA best practice care guideline(s) for all institutions: *“There’s a lot of work…formulating those [Epic Beacon] plans and it would be wonderful if we’re all trying to standardize, including the NCCN guidelines to have that worked out and shared”* [Facilitator, Mixed, 2019]. A leading barrier was complexity of AYA ALL care: *“If you don’t have the clinical ability to see [AYA ALL]… then they’re never going to come to you in the first place”* [Barrier, Physician, 2021].

*Communication & Collaboration* identified facilitation of GCC through multi-disciplinary teams, regularly scheduled team meetings, communication strategies, and pediatric/adult collaborations including sharing eligibility information to aid screening and cross-enrollment on trials: *“For AYA who enter the adult institution first, the compliance with NCCN guidelines falls off pretty quickly… We now have folks embedded in the adult institution, so we’re able to monitor this more closely”* [Barrier, Physician, 2021].

*Provider Education* identified lack of training in pediatric-inspired regimens as a barrier to GCC: *“We didn’t get trained on pediatrically inspired regimens… [it would help to] learn… how they do things, and translate that into local community”* [Barrier, Physician, 2021].

#### 3.2.2. Documentation of GCC

Six main ***themes*** with 11 associated *sub-themes* arose for GCC documentation. ***Care Organization*** was overwhelmingly the most prominent theme across FG types, followed by ***Individuals*** and ***Supportive Care.***

Within ***Care Organization****,* sub-themes of *Electronic Medical Records (EMR)* and *Practice Standards & Expectations* captured most coded statements*. EMR* emerged as a facilitator and barrier for documentation workflows. One participant suggested, *“a checklist of what needs to be… documented [for GCC]”* [Facilitator, Mixed, 2019]. Further, *“sometimes the adult side is not aware of these guidelines as much as the pediatric side… it would be nice… if there is an AYA diagnosed, that the EMR is able to make what’s available as far as protocols for this population”* [Facilitator, Non-Physician, 2021]. For *Practice Standards & Expectations*, notable barriers in top-ranked statements included lack of roadmaps (or not accessible), no documentation templates or consensus on documentation practices: *“if someone’s not on study, particularly, I think the documentation gets a little more lax. And things that are part of the NCCN guidelines might not be included in your daily note”* [Barrier, Physician FG, 2021].Suggested facilitators in ranked statements included strong research staff, use of “dot phrases,” and staff/provider training to standardize documentation.

For ***Individual Factors***, all groups, but particularly physicians, described barriers around *Provider Factors* that impact documentation of GCC. In ranked statements, knowledge of AYA ALL NCCN guidelines and subjective treatment practices influence the likelihood of documenting GCC: *“I will just confess I don’t look at the NCCN guidelines. I regard them as something to look at for something I’m not as familiar with treating. So, in sum, I think just an actual lack of familiarity or awareness of those particular guidelines for ALL”* [Barrier, Physician FG, 2021].

***Supportive Care*** was a leading theme for non-physician and mixed groups, noting potential to facilitate GCC documentation through enhanced patient assistance (e.g. designated navigators, oral medication calendars, and medication education).

## 4. Discussion

This study describes structure- and process-level barriers and facilitators impacting GCC among AYA with ALL, as identified through FG participation and use of nominal process. Themes of ***Care Organization***, ***Supportive Care***, and ***Individuals*** ranked highest for both delivery and documentation of GCC. Findings further identify targetable areas to increase GCC and improve AYA ALL outcomes.

**Figure 3.**
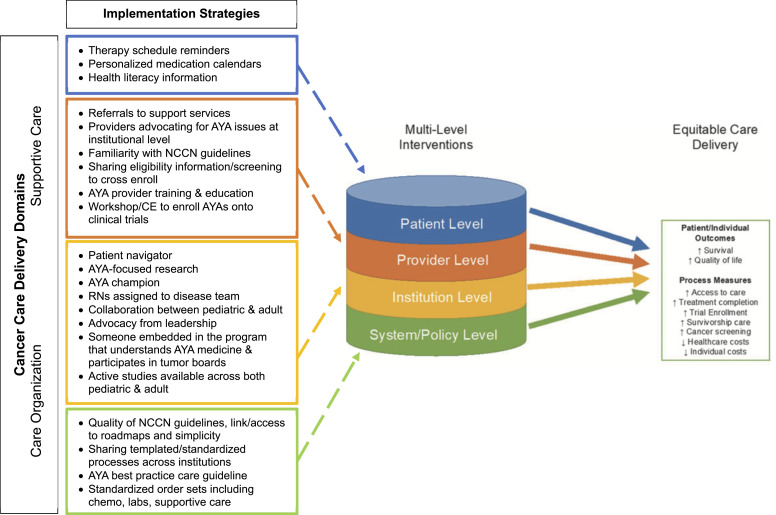
AYA ALL Cancer Care Delivery framework adapted to include NCORP-proposed Specific Multi-Level Implementation Strategies (adapted from Beauchemin M, et al, JAYAO, 2023). Implementation Strategies are derived from ranked statements, listed in full in Table 2

Data support that optimization of care for AYA ALL has no one-size-fits-all solution, and heterogeneity across clinical facilities, ranging from aspects of the health systems and resource environments to multi-disciplinary teams, must be considered when tailoring practical implementation strategies for diverse patients, providers and systems. Identified barriers and facilitators to GCC can be linked to implementation strategies and incorporated into existing frameworks supported by prior NCORP-focused cancer care delivery research,^35^ thus populating a blueprint of actionable next-steps (Figure 3). Example approaches include standardized trainings, bundled toolkits, population-specific checklists and algorithms, and practice protocols, though further work is needed to identify which strategies function best across practice settings, accounting for variation in facility structure/organization, patient population, and practice capacity.

Discussion from these FGs were consistent with findings previously reported by our team, specifically the important role of model of care (adult-only, pediatric-only, mixed) at the CF where care is being delivered.^30^ While many adult-only (60%) and pediatric-only (40%) CFs had an average annual AYA ALL volume of <5/yr, more adult CFs saw ≤10/yr (adult: 94%, pediatric: 73%, mixed: 62%). Nevertheless, in multivariable analysis only facility model was associated with delivery of guideline-concordant care (adult vs. ped+mixed: OR=0.02, 95%CI, 0.00-0.18) while AYA ALL volume was not associated with guideline-concordant care.^36^ Pediatric and mixed CFs also reported more interaction between pediatric/adult oncology teams (tumor boards, initiating contact, or having an established counterpart) than adult-only practices.^30^ It may follow then that limited interactions or collaboration opportunities between pediatric and adult oncology in adult-only settings further challenge pediatric-inspired treatment regimens which may be unfamiliar.

Many barriers and facilitators described during FGs directly support evidence-based strategies as process measures that can improve implementation of GCC, reducing AYA ALL outcome disparities. Since these specific strategies function within components of a healthcare delivery model, it is imperative to study them in a multi-level context. Adapting the published AYA cancer care disparities framework,^35^ these strategies can be grouped by patient-, provider-, institution-, and system/policy-level (Figure 3).

Multi-level implementation strategies were identified using exploratory qualitative inquiry that leveraged the expertise of diverse healthcare professionals, providing evidence to support improvement in GCC as a sustainable intervention for AYA ALL. Across FG themes, specific patient-level strategies included local housing access for out-of-town AYA, personalized medication calendars and treatment reminders, health literacy information, and involvement from a caregiver. Concrete provider-directed strategies elicited in FGs included AYA-specific education, hands-on training, provider advocacy for AYA, recurring team meetings to improve communication, and opportunities for collaboration between adult and pediatric providers like COG members for medical oncologists. Institution-level implementation strategies included identification of AYA champions and psychosocial support staff, pharmacy engagement, pediatric and adult collaborations, AYA advocacy from cancer center leadership, and open-sharing of roadmaps and eligibility information to aid clinical trial cross-enrollment. Additional pragmatic tools included standardized order sets that bundle treatment, labs, and supportive care, along with institutional guidelines to dictate location of care for AYA with an established referral process. Specifically, physician FGs highlighted the “complexity of care for the patient population” when discussing these strategies in support for GCC for AYA ALL. Finally, notable strategies addressing system- or policy-level factors included prioritizing AYA-focused research and creating pathways for sharing standardized/templated processes for AYA cancer care across institutions, including a national AYA best practice care guideline. Opportunities to improve NCCN guidelines themselves were suggested, like providing roadmaps and other materials for recommended regimens to enhance accessibility and navigation. As guideline content continues to evolve (2025 NCCN ALL guidelines specify pediatric-inspired therapy for AYAs without significant comorbidities^37^; 2026 NCCN AYA guidelines reference ALL guidelines for therapy). Of note, the American Society of Hematology recently issued guidelines regarding frontline therapy for AYAs with ALL, with a recommendation for pediatric-inspired therapy along with supportive care recommendations.^38^ Whether these address some of the FG suggestions warrants further examination.

The potential application of these findings reaches far beyond the NCORP. Capacity for AYA cancer care varies greatly across community-based practices. Recent assessments of diverse practice settings can improve generalizability of findings, providing further insight regarding resource limitations that may impact both AYA access to cancer care and the feasibility of strategies to improve GCC. Among 271 practices participating in the 2022 NCORP Landscape Assessment, 37% met one of the AYA cancer care capacity criteria (self-identify as having an AYA Program, treat ≥50 AYAs with cancer/year, and/or AYA comprising ≥5% of annual cases), of which only 20% reported having AYA-specific resources.^39^ Among the 84 practices participating in ACCL16N1CD that saw ≥1 AYA with ALL/year, most (79%) had no AYA-specific resources.^30^ However, AYA capacity remains limited outside community settings also; even among NCI-designated Comprehensive Cancer Centers, only half report having AYA oncology programs.^40^ Future AYA care delivery studies across NCORP and non-NCORP practices should incorporate an inclusive up-front approach with scalable implementation and dissemination strategies.

These findings must be considered in the context of study limitations. FG data represent a fraction of healthcare professionals caring for AYA ALL; however, diverse sampling across NCORP characteristics and roles sought to optimize representative participation of practice settings. Also, two different participants joined in two FGs that were two years apart, though any redundancy was likely offset by NGT procedures and achieving saturation of ideas across FG types. The timing of FGs began prior to the COVID-19 pandemic (2019) and continued through peak pandemic years (2023) which may have placed unknown stressors on practice settings and healthcare professionals impacting volunteerism; on the other hand, shifting from an in-person to virtual format may have enhanced accessibility for professionals from some practices. In addition, while healthcare professionals across participating NCORP practices were invited to participate, the distribution of FG volunteers (adult: 17%, pediatric: 43%, mixed: 41%) may limit the contribution of unique experiences faced in adult-only settings related to pediatric-inspired therapy. That being said, the voice from mixed CFs is important to include in that while more participating CFs were adult (adult: 55%, pediatric: 17%, mixed: 28%), with substantial variation in annual volume of AYAs with ALL (very low at most models), more AYAs were treated at mixed CFs (adult: 30.4%, pediatric: 29.5%, mixed: 40.1%).

## 5. Conclusion

This study identified barriers and facilitators related to themes of Supportive Care and Care Organization that can impact the proportion of AYA with ALL receiving treatment according to NCCN guidelines. Implementation strategies derived from FGs of healthcare professionals caring for AYA ALL at NCORPs provide guidance for future care delivery studies with the potential to inform institutional and national changes to practice and policy. Ultimately, these findings inform an intervention roadmap with actionable steps for implementation toward providing equitable, high-quality, evidence-based care to all AYA with ALL.

## Supplemental Material

Supplemental Material - Adolescents and Young Adults With Acute Lymphoblastic Leukemia: Qualitative Barriers and Facilitators to Guideline-Concordant Care Supplemental Material for Adolescents and Young Adults With Acute Lymphoblastic Leukemia: Qualitative Barriers and Facilitators to Guideline-Concordant Care by Allison C. Grimes, MD, MSCI, Julie A. Wolfson, MD, MSHS, Charlotte L. Kerber, MS, Kristin M. Bingen, PhD, Melissa P. Beauchemin, PhD, RN, CPNP-PC, CPON, Koh B. Boayue, MD, Jennifer M. Levine, MD, MSW, Michele A. Scialla, MSN, RN, CCRC, Wendy L. Woods, MD, MPH, Jane J. Liu, MD, Olivia Ponce, BA, Sarah L. Vargas, PhD, Anjali S. Advani, MD, George J. Chang, MD, MS, MHCM, Emily K. Curran, MD, Dawn L. Hershman, MD, MS, FASCO, Selina M. Luger, MD, Kristen M. O’Dwyer, MD, Wendy Stock, MD, David S. Dickens, MD, Michael E. Roth, MD, David R. Freyer, DO, MS, Brad H. Pollock, PhD, MPH, Lillian Sung, MD, PhD, Susan K. Parsons, MD, MRP in Cancer Control.
