# Supplementary material for: Adolescents and Young Adults With Acute Lymphoblastic Leukemia: Qualitative Barriers and Facilitators to Guideline-Concordant Care
Source: Cancer Control. 2026 Jul 20;33:10732748261469238. doi: 10.1177/10732748261469238 (PMC13385608; doi:10.1177/10732748261469238)
Supplement: Supplemental Material - Adolescents and Young Adults With Acute Lymphoblastic Leukemia: Qualitative Barriers and Facilitators to Guideline-Concordant Care [file sj-pdf-1-ccx-10.1177_10732748261469238.pdf]

APPENDIX I
RECRUITMENT EMAIL

Dear NCORP Team,

Thank you again for opening ACCL16N1CD at your NCORP/site! As part of Aim 3 of the protocol, we are inviting health care providers from your site to volunteer to participate in study focus groups – **these are being held in a VIRTUAL format, and each group is expected to last no longer than 90 minutes**.

At this time, eligible participants include all physicians who are employed at your site (e.g., not fellows) and who provide direct care to patients 15-39 years of age (adolescents and/or young adults: AYA) with Acute Lymphoblastic Leukemia (ALL). We enthusiastically welcome all volunteers – adult and pediatric specialists alike – regardless of how few AYA ALL patients they may treat.

It is important that all eligible physicians at your site (to the best of your ability) are sent this invitation to ensure that we don't have a biased sample. See protocol Section 3.6 for details.

Volunteers selected to participate in this focus group will be extended an invitation. **Since all focus groups will take place online, all selected participants must have a reliable internet connection**, **access to an audio/video-capable device. If possible, access to dual screens** (either a dual screen computer set-up or a desktop/laptop plus second laptop/iPad device for use during the session) would also be ideal. Detailed instructions for virtual participation will be sent to all participants ahead of the scheduled focus group.

Please send the message below to all eligible physicians at your site. The email **contains a link to a brief volunteer survey** - please encourage them to complete it **within 1 week of receiving the invitation as some of the open slots for focus groups are quickly approaching**. We also ask that you discuss the importance of the study with your staff.

Thank you for facilitating ACCL16N1CD! Here is the e-mail we suggest that you send (includes the survey link):

> Hello,
>
> Your institution is participating in **ACCL16N1CD, *Documentation and Delivery of Guideline-Consistent Treatment in Adolescent and Young Adult (AYA) Acute Lymphoblastic Leukemia (ALL).*** This study focuses on AYA ALL guidelines, with Aim 3 looking at facilitators of and barriers to their clinical use among patients 15-39 years of age. You are now invited to nominate yourself for consideration for participation in this study.
>
> **We enthusiastically welcome physicians to volunteer who provide direct care to AYA patients 15-39 years of age with ALL - adult and pediatric specialists alike – regardless of how few AYA ALL patients they may treat.**
>
> If you nominate yourself for participation in Aim 3 of this study and are selected to participate, you may be invited to attend a **virtual focus group** (hosted via GoToMeeting). We anticipate that each focus group session will last under 1.5 hours.
>
> The focus group moderator will ask about your perceptions of the use of AYA ALL NCCN guidelines at your site and ask the group to discuss factors which may help or hinder the implementation of such guidelines. Groups will be separated based on the background and clinical roles of the participants (physician groups and non-physician groups). Participants may only participate in one focus group.

Your participation in this study is completely voluntary. Even if you nominate yourself for participation in this study, you may not be selected for participation. You may change your mind about participating at any time before the focus group begins or during the focus group discussion.

Please contact any member of the study committee or your NCORP PI if you have questions.

If you wish to nominate yourself for consideration for participation in a focus group, please complete this brief online survey: [LINK]

If you have any questions, please email: ACCL16N1CDinfo@childrensoncologygroup.org.

Thank you for considering joining ACCL16N1CD. We are very much looking forward to working with you soon!

On behalf of the ACCL16N1CD study committee,

Julie Wolfson, ACCL16N1CD Study Chair (jwolfson@uabmc.edu)
Allison Grimes, ACCL16N1CD Study Vice Chair (grimesa@uthscsa.edu)
Emily Curran, Alliance Study Champion (curraney@ucmail.uc.edu)
Wendy Stock, Alliance Study Champion (wstock@medicine.bsd.uchicago.edu)
Jane Liu, Alliance Community Oncology Study Champion (JLiu@illinoiscancercare.com)
Anjali Advani, SWOG Study Champion (ADVANIA@ccf.org)
Kristen O'Dwyer, SWOG Study Champion (Kristen_Odwyer@URMC.Rochester.edu)
Selina Luger, ECOG/ACRIN Study Champion (Selina.Luger@uphs.upenn.edu)

APPENDIX II
VOLUNTEER FORM

ACCL16N1CD Aim 3 Questionnaire

By completing this questionnaire, you are indicating that you wish to be considered for participation in ACCL16N1CD by attending a focus group discussion. Participation is entirely voluntary.

Your name and contact information are needed for focus group organization and logistics, however this information will not be included in the study database.

1.) Full Name: ____
Last Name First Name

2.) Affiliated Institution: ____

3.) Email Address: ____

4.) Phone Number that we may reach you at: ____________________________________

5.) Are you a current trainee? (*Please note trainees are not eligible to participate in this study. If you are a current trainee, please do not complete the rest of the questionnaire)
[ ] Yes
[ ] No

6.) Please specify the specific type of health care professional you are (select one):
[ ] Clinical Research Associate
[ ] Nurse Practitioner
[ ] Patient Advocate
[ ] Pharmacist
[ ] Physician
[ ] Physician Assistant
[ ] RN
[ ] Social Worker
[ ] Other (please specify): ____

7.) How many years of experience do you have in pediatric oncology, including pediatric oncology specific training?
[ ] [ ] years [ ] [ ] months

8.) Please provide an estimate of the number of AYA patients that are treated under your care annually.
[ ] 1-5
[ ] 5-10
[ ] 10-25
[ ] 25+

9.) Please provide an estimate of the number of ALL patients that are treated under your care annually.

[ ] 1-5
[ ] 5-10
[ ] 10-25
[ ] 25+

10.) Please select all professional meetings which you attend annually.

ACCP

Alliance

APHON

ASPHO

COG

ECOG-ACRIN

ONS

SWOG

Other (please list): ______________________________

11.) As a percentage, what is the proportion of time you spend devoted to direct patient care? (For example: an employee working 40 hours weekly who spends 20 hours of that time engaged in direct patient care would answer 50%.)

[ ] [ ] %

12.) Please select which hospital type best describes your NCORP institution:

[ ] Stand-alone Children's Hospital
[ ] Pediatric program embedded within an adult hospital
[ ] Adult hospital

13.) Please select which setting applies to your NCORP institution:

[ ] Academic program
[ ] Non-academic program

14.) Which term best describes the location of your NCORP institution:

[ ] Rural
[ ] Urban

Thank you for your willingness to participate in ACCL16N1CD!
If you are selected to join a focus group, we will contact you by e-mail to confirm your willingness to participate and to make arrangements.

DEMOGRAPHIC QUESTIONNAIRE FOR AIM 3 PARTICIPANTS

The information requested below is being collected for the purposes of NIH inclusion enrollment reporting.

1. Full Name: ____
   Last Name First Name

2. Gender: [ ] Female [ ] Male

3. Race:
   [ ] American Indian or Alaska Native
   [ ] Asian
   [ ] Black or African American
   [ ] Caucasian or White
   [ ] Native Hawaiian or Other Pacific Islander
   [ ] Unknown

4. Ethnicity:
   [ ] Hispanic or Latino
   [ ] Not Hispanic or Latino
   [ ] Unknown

APPENDIX III
MODERATOR GUIDE

Introduction

Today we will be discussing the NCCN guidelines for AYAs with ALL. [SLIDE] These comprehensive guidelines detail the management and interventions of 97% of cancers in the US and are based on the best available evidence at the time they're derived. They are continuously and rapidly updated as new data and clinical information become available.

For example, [SLIDE] the Guidelines are available for free online, simply by creating a free account. The NCI considers an AYA to be someone who was diagnosed with cancer between the ages of 15 and 39 years, and the NCCN guidelines for AYA echo this age-based definition of AYA. By following the link to Guidelines for Special Populations, there are Guidelines available for professionals, for patients, or a quick guide for patients. Once in the AYA Guidelines, [SLIDE] there are only a few diagnoses with disease-specific guidelines related to therapy, and these links lead us to the disease-specific guidelines. Looking at these in detail for ALL, [SLIDE] we see the specific attention to in patients 15-39 (here we are showing the Ph- guidelines). The guidelines are laid out here for induction and consolidation, where we see the recommendation at this point in time for a clinical trial or pediatric-inspired multi-agent chemotherapy. When we look at the footnote to this, we see the list of pediatric-inspired protocols for AYA patients.

Although the guidelines are updated regularly, the recommendations we show here were in place during our retrospective study period (i.e. 2012 to 2016). However, for a number of reasons, treatment of ALL in AYAs varies greatly across clinical settings.

In our discussion today, will be talking about what factors may influence the treatment of AYAs with ALL at NCORP sites. Specifically, we will talk about what may serve as a barrier or a facilitator to the **documentation** and the **delivery** of AYA ALL treatment consistent with these guidelines. **For the sake of this discussion, we will not be focusing on any particular guideline from any particular timeframe, rather we are most interested in what barriers and facilitators may exist to delivering AYA ALL treatment consistent with NCCN guidelines in general.** First, what is a barrier? What is a facilitator? [SLIDE]

We will be using nominal group technique, which is an approach that helps ensure that everyone's opinion is heard. To be fully transparent, here are some ground rules for our discussion. [SLIDE]

I have 4 questions. [SLIDE] First, I am going to pose question #1 to the entire group and ask each of you to think of your responses in silence. You are encouraged to write your responses down. Next, we will ask each of you to share your responses, which we will write and display. We will continue this until there are no more responses. If you don't have anything new to add, you can skip your turn and we'll go to the next person. Then, we will discuss all of these responses as a group to make sure that they are clear.

Once each of you has shared at least one response to the first question, and we are comfortable as a group that the responses are clear, it will be time for you to rank-vote.

As you look at each of the responses, you will rank them according to which is most important to you. We will then view how people have ranked each of the items and then freely discuss the overall rankings.

After discussing, everyone will then vote again by ranking which of the responses are most important. Once again, a set of overall rankings will be generated.

We will repeat this same process for subsequent questions.

Question #1 [SLIDE]

1) **Please think about your answer to the following question silently:**

Thinking about how treatment is delivered in routine practice at your NCORP institution: what are the most important **barriers** to **delivering** NCCN-concordant therapy for AYAs with ALL?

2) **Now, take 3 minutes to silently think about this question and jot down your ideas.**

3) **Please share your responses! Let's go around the group. If all your points have been raised or if your point is similar to one that has already been raised, feel free to 'pass'.**

    Moderator will alternate each round starting with a different participant. Moderator guides conversation so that each focus group member offers barriers until all thoughts are exhausted. Moderator will allow minimum of 2 times around for minimum of 2 responses per participant, may be cut for time after that point.

4) **Let's review the list of items that we have generated. The number of items doesn't matter – we can have as many or as few as you want. But…**
    - **Are any so similar that they can be combined without losing any meaning?**
    - **Should any one of the items be split into more than 1 idea?**
    - **Is there anything missing?**

    Moderator can probe here to flush out details of items. Suggested Probes:
    - *Are NCCN guidelines currently incorporated in your practice to direct patient care? If so, how are they incorporated? Can you provide an example?*
    - *… when caring for patients? discussing care with colleagues? teaching?*

5) **Let's each silently re-read what you've identified as barriers to delivering therapy for AYAs with ALL that is consistent with NCCN Guidelines. Is there anything else anyone would like to share about this particular treatment at their institution?**

VOTE #1.1 [SLIDE]

6) **Now you have the opportunity to vote by ranking the responses which are most important to you. Once you are done, please wait for others to finish.**

7) **Let's review your votes.**

    Moderator reads out items in rank order.

8) **So, it seems like you are saying that [item], [item] and [item] are important to this group. Let's discuss some reasons for your selections. Which items are most important to you and why? Remember that it is fine to disagree and that there is no right answer.**

    Moderator guides discussion regarding reasons for rankings, by going through items or down the participant list to ensure each participant has an opportunity to share ideas.

9) **Does this ranking reflect your personal experience?**

VOTE #1.2 [SLIDE]

10) **Now you have the opportunity to vote again by ranking the responses which are most important to you. You may vote the same as before or take the opportunity to vote differently if your opinions have changed. Once you are done, please wait for others to finish.**

Now it's time to move onto the next question.

Moderator will repeat steps 1 through 10 for each subsequent question.

Question #2 Thinking about how treatment is delivered in routine practice at your NCORP institution: what are the most important **facilitators** to **delivering** NCCN-concordant therapy for AYAs with ALL?

Question #3 Thinking about how treatment is delivered in routine practice at your NCORP institution: what are the most important **barriers** to **documenting** NCCN-concordant therapy for AYAs with ALL?

Question #4 Thinking about how treatment is delivered in routine practice at your NCORP institution: what are the most important **facilitators** to **documenting** NCCN-concordant therapy for AYAs with ALL?

[SLIDE] Thank you! We've just talked a lot about some factors that might help or hinder delivery and documentation of treatment for AYAs with ALL in concordance with NCCN guidelines. We will summarize all your ideas and comments, bring them together with those that are generated by other focus groups and will use this information to develop trials to look at the impact of specific strategies to enhance treatment of this population according to NCCN guidelines.

This is the end of our session. **You have been a wonderful focus group!** We would welcome any comments you have about your experience today or suggestions for improvement. Thank you all very much for your work today and for your enthusiasm!

APPENDIX IV
CODE BOOK

This codebook provides inclusion and some exclusion criteria for domains included as part of healthcare-level factors in the conceptual framework. This codebook includes *preliminary* definitions and coding criteria; we may include other information, like examples of coded text.

General Coding Rules: When two codes are in question for a passage, consider the primary meaning of the passage to assign code; consider what the participant is truly saying. Analysts may wish to err on the side of inclusion or double coding.

**I. Structure**	
**A. Hospital Type**	Designation as a either a stand-alone children's hospital, adult-only hospital, or pediatrics embedded in a general hospital (i.e. mixed).
NCI Designation	NCI-recognized centers that meet rigorous standards for transdisciplinary, state-of-the-art research focused on developing new and better approaches to preventing, diagnosing, and treating cancer; includes NCI-Designated Cancer Centers and Comprehensive Cancer Centers. Include statements about NCI designation and extent to which interviewees view its influence on cancer care delivery, e.g., comprehensiveness of care, including enhanced supportive care and psychosocial support; access to clinical trials and/or clinicians practicing adjacent to trials. Exclude or double code statements related to affiliation with university medical centers and institutions that engage only in cancer research.
Facility Volume	Volume of patients may be captured in number per unit time (month, year, etc.) or number of new cancer patients/year or specifically volume of acute lymphoblastic leukemia (ALL) cases or number of adolescent and young adult (AYA) cancer cases/year. Volume may also reference number of AYA ALL cases/year.
Academic Status	References made to academic affiliation/accreditation and/or relationship to medical school/campus.
Single vs. Multi Location	Reference number of distinct practice locations (single=1, multi≥2).
**B. Model of Care**	Clinical practice structure and culture around age and/or age group; paternalistic (pediatric) vs. individualistic (adult) models. Include statements associated with treatment within a pediatric model of care vs. an adult/internal medicine model); provider specialty; age restrictions; unmeasured facility characteristics contributing to these model differences (children's hospital vs. other); NCORP structure; AYA referral patterns/source; AYA institutional guideline dictating location of care.
**II. Process**	
**A. Care Organization**	Complexity of care and coordination of related services inherent to provision of clinical care for AYA ALL. Includes set-up and ease of access to laboratory services, referral structures, guidelines, trainings, existing networks or established communication systems, processes for information sharing, standardized orders, and team-based organizational factors including clinic flows, types of scheduled meetings, leadership structure, types of team members

	and roles.
Practice Standards/Expectations	Policies, guidelines (written or unwritten), and care practices that are considered guides or standards in how care is provided or documented.
Electronic Medical Record	Includes statements that reference the role of the EMR in influencing documentation or delivery of AYA ALL therapy.
Time allocation	Time taken (protected or unprotected) to document or deliver care
External partnerships/ collaborations	Higher-level organization/centralization of practice through external networks, partnerships, formal communications or memberships; includes best practice standards, information sharing and access.
Communication/ collaboration	Collaboration by physicians and staff across medical/pediatric oncology and with community providers. Include statements that describe team evaluation rooted in transdisciplinary physician/staff collaboration; use of novel (pediatric vs adult) therapy; physician training (location, era, specialty, subspecialty, etc.) or physician practice (proportion of practice devoted to clinical practice, academic nature of practice); includes internal collaborative processes to promote trial cross-enrollment.
Provider education	Includes provider training, workshops, familiarity with AYA-specific needs and guidelines.
Resource allocation	How resources are distributed across an institution.
**B. Supportive Care**	Support structure, resources, and access to comprehensive supportive care services that complement the treatment experience for AYA ALL regarding the mental, emotional, physical, and economic burdens of therapy. Includes support and access to psychosocial care, care navigation, assessments and interventions for social determinants of health, and philanthropic, financial and insurance support.
Psychosocial Support	Support for clinically significant psychological distress and adverse mental health outcomes that can continue into posttreatment survivorship. Unmet needs as predictors of distress, including informational, counseling, practical support, legal, childcare, and educational/vocational needs. Include statements that describe collaborative engagement among multidisciplinary teams and key stakeholders, developmentally appropriate care, needs assessments, and workflow integration connecting AYAs and appropriate psychosocial services. Include specific reference to psychologist or social worker. Include fertility preservation referrals and practices preserving the capacity to have biological children with gender-specific recommendations. Include statements that describe sperm banking and embryo, oocyte, and ovarian tissue cryopreservation, standard of care and procedural costs. Exclude or double code statements related to sexual dysfunction.
Patient Education	Include treatment education, health literacy, patient-directed languag
AYA Services	Support services where AYAs are treated by a facility/oncologist knowledgeable in AYAs. There is no set of minimal services, standards, or metrics by which institutions consider their approach to be an AYA program. Include statements that describe HRQoL related to being treated at a site with or without a formal AYA program; program forms including psychosocial support or medical

	and psychosocial support, only one or many disease groups; AYA advocacy and institutional support; AYA champion. Includes statements related to local AYA-focused research. Exclude or double code statements related to psychosocial support, fertility preservation, survivorship care.
Treatment adherence	Captures patient adherence to medications, clinical appointments, hospital visits for therapy, and instructions related to therapy.
**C. Therapeutic Approach**	Treatment style or approach to determine treatment for AYA ALL. Includes planning for up-front therapy and management of ongoing therapy including decision-making based on age, treatment-related toxicity, trial access, therapy guidance, and institutional support impacting treatment decisions.
Clinical Trial Enrollment	The process of active evaluation of potential participants for enrollment in a trial. Include statements that describe trial availability, enrollment patterns at treating institutions, and regulatory needs due to facility structure; upper age limits of either pediatric trials or children's hospitals; cross-network studies developed jointly by pediatric/adult principal investigators; activation via the Cancer Trials Support Unit (CTSU) by pediatric/adult cooperative groups. Consider if institutional membership in cooperative groups should be double coded for structure and process (bidirectionality between hospital type and care model).
Treatment Regimen	Pediatric and adult oncology backbone approaches to therapy that may vary (either significantly or subtly) in many malignancies that also occur in AYAs. Include statements that describe joint trials between the adult and pediatric National Clinical Trials Network (NCTN) cooperative groups; comparison of these different approaches among AYA; best practices.
Treatment-Related Toxicities	Biologically-driven inferior AYA outcomes that lead to more intensified treatment regimens, and thus more adverse toxicity profiles. Include statements that describe differential metabolism of certain agents, developmental stage (when treatment is delivered) and associated hormonal differences impacting metabolism, or a different risk profile; increased infection-related mortality; availability of supportive care for toxicities.
**III. Additional Codes**	
**A. Individual Factors**	Specific characteristics, behaviors, or practices at the individual level (including provider or patient) that either positively or negatively influence acceptability of, access to, perspectives on, or implementation of guideline-concordant AYA ALL therapy.
Patient Factors	Includes insurance status, socioeconomic status, financial challenges, culture, social issues, geographic challenges, transition to age of majority, parent or caregiver involvement, work or school constraints, family constraints, and adherence factors.
Provider Factors	Includes personal knowledge gaps of oncology providers surrounding AYA population, AYA ALL therapy approaches, or awareness of age-specific guidelines; also includes gaps in knowledge/awareness of referring providers.

APPENDIX V
DATA SUMMARY TABLE

A. Barriers to Delivery

FG No.	FG type	Year	Rank	Statement	Theme(s) and Sub-theme(s)
1	Mixed	2019	1	Inability to give subsequent therapies due to treatment toxicity/AEs of drugs/infection.	Therapeutic Approach<br>Treatment-Related Toxicities
1	Mixed	2019	2	AYA referral process/source (adult PCP to adult oncology vs. pediatric PCP to pediatric oncology); inconsistent placement of patient.	Model of Care
1	Mixed	2019	3	Increased adherence issues in AYA (e.g., medications).	Supportive Care<br>AYA Services<br>Individuals<br>Patient Factors<br>Treatment Adherence
1	Mixed	2019	4	Social and cultural issues impact treatment decisions (including geographic/travel challenge).	Individuals<br>Patient Factors
1	Mixed	2019	5	Complexity of AYA pediatric backbone protocols.	Therapeutic Approach<br>Treatment Regimen
2	Non	2019	1	Coordinating treatment with patient schedules as AYAs have work, families, and are less flexible.	Individuals<br>Patient Factors<br>Supportive Care<br>Psychosocial Support
2	Non	2019	2	Concerns about patient compliance.	Individuals<br>Patient Factors<br>Supportive Care<br>Treatment Adherence
2	Non	2019	3	Complexity of protocols/publications without adequate guidance.	Therapeutic Approach
2	Non	2019	4	Higher cumulative doses in pediatric protocol, concern for increased toxicities.	Therapeutic Approach
2	Non	2019	5	Institutional resources, more is needed for pediatric regimens.	Therapeutic Approach<br>Care Organization<br>Resource Allocation
3	Mixed	2019	1	Poor adherence to medications/appointments.	Supportive Care<br>Treatment Adherence<br>Individuals<br>Patient Factors
3	Mixed	2019	2	AYAs are underinsured or have no insurance, also lower socioeconomic status.	Individuals<br>Patient Factors
3	Mixed	2019	3	Low clinical trial access/availability for AYA (especially COG or non-COG sites).	Therapeutic Approach<br>Clinical Trial Enrollment
3	Mixed	2019	4	Poor communication between pediatric and adult providers and community providers.	Care Organization<br>Communication & Collaboration
3	Mixed	2019	5	Transition to age of majority on therapy leads to challenges with buy-in/consent/adherence.	Individuals<br>Patient Factors
4	Non	2021	1	Compliance of this age group (taking medications, reporting side effects, waiting until very sick before asking for help).	Supportive Care<br>Treatment Adherence
4	Non	2021	2	Financial issues and lack of support interfere with care, ability to take time off, transportation, etc.	Individuals<br>Patient Factors
4	Non	2021	3	Poor health literacy impacts information provided explaining treatment,	Supportive Care

FG No.	FG type	Year	Rank	Statement	Theme(s) and Sub-theme(s)
				understandable language, fertility issues in this age group.	Patient Education<br>Individuals<br>Patient Factors
4	Non	2021	4	Determination of where a particular AYA will "fit in" for treatment (pediatric vs. adult).	Model of Care
4	Non	2021	5	Gap in research studies available for AYA population, may not be eligible for clinical trial based on age.	Therapeutic Approach<br>Clinical Trial Enrollment
5	Phys	2021	1	Limitations/knowledge gap in local community-based providers leading to reduced referrals to cancer center for ALL (provider-dependent).	Individuals<br>Provider Factors
5	Phys	2021	2	Age restrictions (younger AYA treated at pediatric site vs older AYA treated at adult site with differences in care/access/assessment).	Model of Care
5	Phys	2021	3	Training deficiencies in pediatric-inspired regimens for adult oncology related to rarity of disease/reduced exposure to ALL leads to gap in knowledge (need for workshop, CE).	Care Organization<br>Provider Education
5	Phys	2021	4	Socioeconomic discrepancies.	Individuals<br>Patient Factors
5	Phys	2021	5	Challenges in obtaining preferred laboratory/molecular sequencing assessments (including requiring case-by-case authorization).	Care Organization
6	Phys	2021	1	Complexity of care for patient population presents challenges in getting patients easily in and out of clinic.	Care Organization
6	Phys	2021	2	Insurance status dictates location of care in some cases and drug coverage.	Individuals<br>Patient Factors
6	Phys	2021	3	Institutional restrictions on age limitations that can be seen at certain locations.	Model of Care
6	Phys	2021	4	Some subspecialists won't see patients over age 18 at stand-alone children's hospital.	Model of Care
6	Phys	2021	5	Social workers, navigators and support staff may be less familiar with AYA specific needs/resources.	Care Organization<br>Provider Education

B. Facilitators to Delivery

FG No.	FG type	Year	Rank	Statement	Theme(s) and Sub-theme(s)
1	Mixed	2019	1	Oncology team approach to AYA care including navigator/coordinator (including patient follow-up, phone calls, education, communication established).	Supportive Care<br>AYA Services
1	Mixed	2019	2	Psychosocial support (travel, insurance, meals, bills, etc.).	Supportive Care<br>Psychosocial Support
1	Mixed	2019	3	Parent or caregiver involvement.	Individuals<br>Patient Factors
1	Mixed	2019	4	AYA best practice care guideline for all institutions to follow.	Care Organization<br>External Partnerships & Collaborations
1	Mixed	2019	5	Having an institutional guideline in place for which AYAs go to pediatric vs. adult oncology.	Model of Care<br>Care Organization<br>Practice Standards & Expectations
2	Non	2019	1	Open-sharing of roadmaps from protocols (e.g., COG) chemotherapy, labs, etc.	Care Organization<br>External Partnerships & Collaborations
2	Non	2019	2	Regularly scheduled team meetings for communication.	Care Organization<br>Communication & Collaboration
2	Non	2019	3	Multi-disciplinary team helps to provide all types of needed care.	Care Organization

FG No.	FG type	Year	Rank	Statement	Theme(s) and Sub-theme(s)
					Communication & Collaboration<br>Supportive Care
2	Non	2019	4	Building relationships with AYA - counselor especially for AYA.	Supportive Care<br>AYA Services<br>Psychosocial Support
2	Non	2019	5	Hands-on training at pediatric institution/resources and networking.	Care Organization<br>Provider Education
3	Mixed	2019	1	Standardized order sets (treatment plan) including chemotherapy, labs, supportive care (guideline-based).	Care Organization<br>Practice Standards & Expectations
3	Mixed	2019	2	Dedicated oncology social worker.	Supportive Care<br>Psychosocial Support
3	Mixed	2019	3	Sharing templated/standardized processes across institutions.	External Partnerships & Collaborations<br>Care Organization
3	Mixed	2019	4	High capacity/engaged pharmacist.	Supportive Care
3	Mixed	2019	5	Philanthropy (to support staff, programming), access to local housing/support for out-of-town AYA.	Supportive Care<br>Psychosocial Support
4	Non	2021	1	Transparency with patients, therapy schedule reminders, how to prepare for visits, personalized medication calendars.	Supportive Care<br>Psychosocial Support
4	Non	2021	2	Rapport with and accessibility of the medical team, including psychosocial.	Supportive Care<br>Psychosocial Support
4	Non	2021	3	Communication between doctors and research staff across pediatric and adult units, enhanced by EMR, easy to access records.	Care Organization<br>Communication & Collaboration<br>Electronic Medical Record (EMR)
4	Non	2021	4	Parental support (the more involved the parents, the more compliant the AYA).	Individuals<br>Patient Factors
4	Non	2021	5	Accessibility to resources (financial, insurance).	Supportive Care<br>Psychosocial Support
5	Phys	2021	1	AYA focused research.	Supportive Care<br>AYA Services
5	Phys	2021	2	Front-line NPs/APNs and CRAs, having an AYA champion, RNs assigned to disease team (i.e. leukemia).	Supportive Care<br>AYA Services
5	Phys	2021	3	Collaboration between pediatric and adult teams; having someone embedded in the program that understands AYA medicine and participates in tumor boards (local/external).	Care Organization<br>Communication & Collaboration<br>Supportive Care<br>AYA Services
5	Phys	2021	4	Advocacy from leadership (i.e. cancer center leader), prioritizing AYA.	Care Organization<br>Supportive Care<br>AYA Services
5	Phys	2021	5	Sharing eligibility information/screening to cross enroll AYAs onto clinical trials.	Care Organization<br>Communication & Collaboration<br>Therapeutic Approach<br>Clinical Trial Enrollment
6	Phys	2021	1	Educating providers so that they are more confident/comfortable with AYA care including additional AYA-specific support staff (i.e. AYA social worker).	Supportive Care<br>AYA Services<br>Care Organization<br>Provider Education

FG No.	FG type	Year	Rank	Statement	Theme(s) and Sub-theme(s)
6	Phys	2021	2	Existing collaboration with adult and pediatric oncology including familiarity with NCCN guidelines and active studies available across both pediatric/adult practices.	Care Organization<br>Communication & Collaboration<br>Therapeutic Approach<br>Clinical Trial Enrollment
6	Phys	2021	3	Medical oncologist(s) with COG membership allows for adult oncology to see/enroll patients onto COG studies and access to roadmaps for off-study patients.	Therapeutic Approach<br>Clinical Trial Enrollment
6	Phys	2021	4	Quality of the NCCN guidelines, ease for users, could be made better with link/access to roadmaps for simplicity.	Care Organization<br>External Partnerships & Collaborations
6	Phys	2021	5	Providers advocating for AYA issues at the institutional level (insurance, expanding age seen by sub-specialists or additional access to other sub-specialists, etc.).	Supportive Care<br>AYA Services

C. Barriers to Documentation

FG No.	FG type	Year	Rank	Statement	Theme(s) and Sub-theme(s)
1	Mixed	2019	1	Multiple EMR systems across spectrum of care sites; lack of access by staff (CRA, RN, etc.) that don't communicate; electronic and paper duplicity.	Care Organization<br>Electronic Medical Record (EMR)
1	Mixed	2019	2	Knowing what are elements that need to be documented; variability of what is documented.	Care Organization<br>Practice Standards & Expectations
1	Mixed	2019	3	Multiple areas to document same info in EMR leading to inconsistent data capture; too many places to look for where and/or what to document.	Care Organization<br>Electronic Medical Record (EMR)
1	Mixed	2019	4	Lack of consistent provider documentation; no way to search EMR due to variability.	Individuals<br>Provider Factors<br>Care Organization<br>Electronic Medical Record (EMR)
1	Mixed	2019	5	Time; having adequate time to document.	Care Organization<br>Time Allocation
2	Non	2019	1	Different documentation practices by setting (inpatient/outpatient).	Care Organization<br>Practice Standards & Expectations
2	Non	2019	2	Lack of electronic roadmap for treatment; documentation is not always up to date.	Care Organization<br>Electronic Medical Record (EMR)<br>Practice Standards & Expectations
2	Non	2019	3	Paper vs. electronic documentation in EMR.	Care Organization<br>Electronic Medical Record (EMR)
2	Non	2019	4	Time.	Care Organization<br>Time Allocation
2	Non	2019	5	Hard to find all the information.	Care Organization<br>Practice Standards & Expectations
3	Mixed	2019	1	Paper + EMR combined systems lead to duplication of effort, poor efficiency, and inconsistencies; challenges with varied communication/documentation of home infusions.	Care Organization<br>Electronic Medical Record (EMR)<br>Practice Standards & Expectations
3	Mixed	2019	2	Information in EMR difficult to find/access; completing EMR is time-consuming; there are multiple EMR locations to document AE's.	Care Organization,<br>Electronic Medical Record (EMR),<br>Practice Standards & Expectations
3	Mixed	2019	3	Medication reconciliation is cumbersome and not user-friendly.	Care Organization,<br>Electronic Medical Record (EMR)

FG No.	FG type	Year	Rank	Statement	Theme(s) and Sub-theme(s)
3	Mixed	2019	4	There are varied consent and documentation processes and roles in pediatric vs. adult oncology; increased patient volume in adult oncology drives different processes (RVU-driven).	Model of Care
3	Mixed	2019	5	High workload and high patient-to-staff ratios.	Care Organization
4	Non	2021	1	Difficulty with EMR documentation (i.e. lack of electronic roadmap, challenges reviewing treatment plan, transferring plan to roadmap, lack of info via EMR for open/eligible trials, hard to track changes to treatment, lack of interface between inpt and outpt).	Care Organization<br>Electronic Medical Record (EMR)
4	Non	2021	2	Provider doesn't update information in EMR or roadmap (or days entered incorrectly).	Individuals<br>Provider Factors
4	Non	2021	3	Lack of patient education (including oral medications).	Supportive Care<br>Treatment Adherence
4	Non	2021	4	No roadmap or only hard copy exists.	Care Organization<br>Practice Standards & Expectations
4	Non	2021	5	Long treatment, reluctance of patients to keep timeline of therapy (leads to treatment delays).	Therapeutic Approach<br>Individuals<br>Patient Factors
5	Phys	2021	1	Lack of awareness of disease-specific NCCN guidelines for AYA (i.e. ALL).	Individuals<br>Provider Factors
5	Phys	2021	2	Lack of utilization or resistance of NCCN guidelines.	Individuals<br>Provider Factors
5	Phys	2021	3	Lack of training around NCCN guidelines, especially in pediatric-trainees where less guidelines exist that are pediatric-specific.	Model of Care
5	Phys	2021	4	Lack of familiarity with what is needed in documentation (differences in patients that are/aren't on study; whether NCCN recs are included in documentation).	Care Organization<br>Practice Standards & Expectations<br>Individuals<br>Provider Factors
5	Phys	2021	5	Documentation is time-consuming and difficult to be complete in a busy practice; no existing templates for this guideline.	Care Organization<br>Time Allocation<br>Electronic Medical Record (EMR)
6	Phys	2022	1	Lack of awareness that specific NCCN guidelines exist; lack of trigger in ALL population to consult NCCN guidelines.	Individuals<br>Provider Factors
6	Phys	2022	2	No specific practice standard (no formal template or included in consent) for documenting NCCN therapy, but rather each cycle/phase of therapy which is planned/given.	Care Organization<br>Practice Standards & Expectations
6	Phys	2022	3	Multiple practice locations and providers (including treatment off-site) with differing health record systems and differing familiarity with NCCN guidelines of AYA care.	Hospital Type<br>Single vs. Multi Facility<br>Care Organization<br>Practice Standards & Expectations
6	Phys	2022	4	Documentation of NCCN-concordance in notes less prevalent practice in pediatric oncology (especially in ALL).	Model of Care
6	Phys	2022	5	Multiple locations in the health record to document therapy and unclear who should be responsible for documenting various aspects of therapy.	Care Organization<br>Electronic Medical Record (EMR)

D. Facilitators to Documentation

FG No.	FG type	Year	Rank	Statement	Theme(s) and Sub-theme(s)
1	Mixed	2019	1	Knowing what to document and who documents (standard for documentation);	Care Organization

FG No.	FG type	Year	Rank	Statement	Theme(s) and Sub-theme(s)
				specified button in Beacon.	Electronic Medical Record (EMR)<br>Practice Standards & Expectations
1	Mixed	2019	2	Note templates, dot phrases in EMR.	Care Organization<br>Electronic Medical Record (EMR)
1	Mixed	2019	3	Single location for treatment plan documentation.	Care Organization<br>Practice Standards & Expectations
1	Mixed	2019	4	Designated point person responsible for documenting this information; "AYA Navigator" creates and consistently uses template.	Care Organization<br>Practice Standards & Expectations<br>Supportive Care<br>AYA Services
1	Mixed	2019	5	Utilization of consent for all patients not only study patients.	Care Organization<br>Practice Standards & Expectations
2	Non	2019	1	Standardized approach for AE; does modifications in chart.	Care Organization<br>Practice Standards & Expectations
2	Non	2019	2	Having an EMR.	Care Organization<br>Electronic Medical Record (EMR)
2	Non	2019	3	Treatment plan within EMR (Beacon).	Care Organization<br>Electronic Medical Record (EMR)
2	Non	2019	4	Roadmap that outlines treatment, labs, etc.	Care Organization<br>Practice Standards & Expectations
2	Non	2019	5	One roadmap that "follows" patient.	Care Organization<br>Practice Standards & Expectations
3	Mixed	2019	1	Oncology summary/single location for patient information of diagnosis and treatment.	Care Organization<br>Practice Standards & Expectations
3	Mixed	2019	2	Cooperative groups to work with industry for oncology-specific EMR/universal EMR.	Care Organization<br>Electronic Medical Record (EMR)<br>External Partnerships & Collaborations
3	Mixed	2019	3	AYA working group to create checklist of what needs to be documented on and off trial (build templates, hard-stops, dot phrases and exportable AYA module).	Care Organization<br>Electronic Medical Record (EMR)<br>Supportive Care<br>AYA Services
3	Mixed	2019	4	Share best practices with other institutions (i.e. AYA RI network).	Care Organization<br>External Partnerships & Collaborations
3	Mixed	2019	5	Clinical nurse educator; audits.	Care Organization<br>Practice Standards & Expectations
4	Non	2021	1	Multi-disciplinary team meetings/discussions/huddles for planned upcoming care (treatment, scans, etc.); staff with access to both inpatient and outpatient EMR.	Care Organization<br>Communication & Collaboration<br>Electronic Medical Record (EMR)
4	Non	2021	2	Oral medication calendars for patient each visit (assists with patient education, clarity of medications); patient and parent support; medication reconciliation with patient/family (including providing copy of roadmap to patient); referrals to support services for poor adherence/compliance.	Supportive Care
4	Non	2021	3	Development of roadmaps at site (paper) that reflects treatment protocol; roadmap that follows patient from inpatient to outpatient.	Care Organization<br>Practice Standards & Expectations
4	Non	2021	4	Presence of a patient navigator.	Supportive Care
4	Non	2021	5	SOP to aid with standardized patient care at institution (supportive care, hydration).	Care Organization<br>Practice Standards & Expectations<br>Supportive Care

FG No.	FG type	Year	Rank	Statement	Theme(s) and Sub-theme(s)
5	Phys	2021	1	Standardized implementation of NCCN guidelines in COG protocols.	Care Organization<br>External Partnerships & Collaborations
5	Phys	2021	2	Including guidelines in the "How I Treat" and/or ASH publication series.	Care Organization<br>External Partnerships & Collaborations
5	Phys	2021	3	Consensus among providers around NCCN concordant treatment.	Individuals<br>Provider Factors
5	Phys	2021	4	Additional education for providers and trainees to improve awareness.	Care Organization<br>Provider Education
5	Phys	2021	5	Inclusion of NCCN hyperlinks from COG content.	Care Organization<br>External Partnerships & Collaborations
6	Phys	2022	1	Awareness that the guidelines exist; education/awareness of providers/staff.	Individuals<br>Provider Factors<br>Care Organization<br>Provider Education
6	Phys	2022	2	Practice changes around documentation that includes staff/provider training for this to be standard practice.	Care Organization<br>Provider Education<br>Practice Standards & Expectations
6	Phys	2022	3	Auto-text or dot phrases; add a link in the protocol or add a check box in the consent document to use that mentions "NCCN guideline-based care".	Care Organization<br>Electronic Medical Record (EMR)<br>Practice Standards & Expectations
6	Phys	2022	4	Formal discussion of patient (journal club, tumor board, group review).	Care Organization<br>Communication & Collaboration
6	Phys	2022	5	Having a strong CRA or regulatory/compliance staff (ensures documentation is adequate).	Care Organization<br>Practice Standards & Expectations

Statements were made by focus group participants then clarified and edited using the nominal group technique approach. Abbreviations: Focus group (FG), Physician-only (Phys), Non-physician Only (Non), Electronic Medical Record (EMR), Clinical Research Assistant (CRA), Registered Nurse (RN), National Comprehensive Cancer Network (NCCN) Adolescent and Young Adult (AYA), Acute Lymphoblastic Leukemia (ALL), Adverse Event (AE), Relative Value Unit (RVU), Children’s Oncology Group (COG), American Society of Hematology (ASH), Responsible Investigator (RI), Standard Operating Procedure (SOP), Primary Care Provider (PCP), Continuing Education (CE), Nurse Practitioner (NP), Advanced Practice Nurse (APN).

APPENDIX VI
VOLUNTEER TO PARTICIPANT FLOWCHART

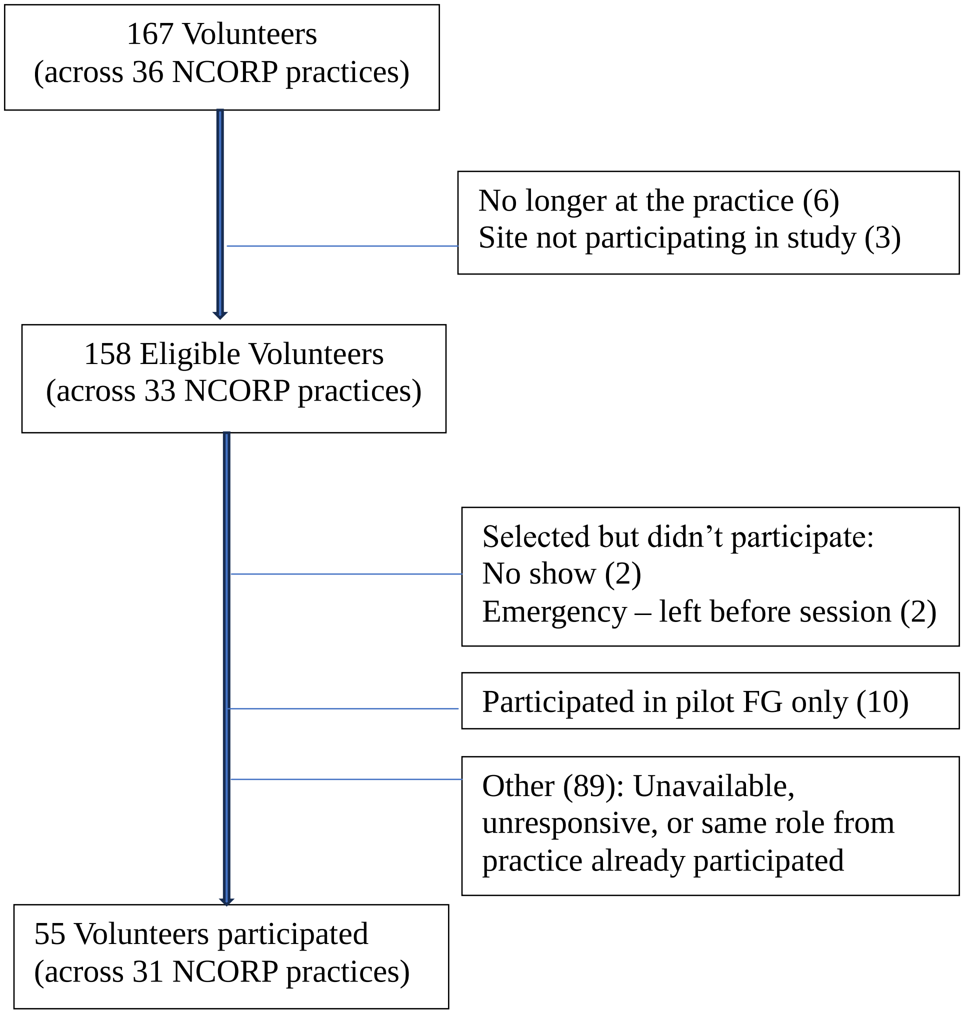

APPENDIX VII
TABLE OF VOLUNTEER CHARACTERISTICS

Eligible Volunteers (n=158)	Adult Only Institution (n=26)	Pediatric Institution (n=68)	Mixed Institution (n=64)	Total
Physician	8/26 (30.8%)	27/68 (39.7%)	29/64 (45.3%)	**64/158 (40.5%)**
Non-Physician	18/26 (69.2%)	41/68 (60.3%)	35/64 (54.7%)	**94/158 (59.5%)**
Nurse (RN)	8/26 (30.8%)	16/68 (23.5%)	20/64 (31.3%)	
Pharmacist	3/26 (11.5%)	2/68 (2.9%)	0	
Nurse Practitioner	4/26 (15.4%)	1/68 (1.5%)	3/64 (4.7%)	
CRA	1/26 (3.8%)	6/68 (8.8%)	2/64 (3.1%)	
Social Worker	1/26 (3.8%)	5/68 (7.4%)	2/64 (3.1%)	
Psychologist	0	3/68 (4.4%)	2/64 (3.1%)	
Other	1/26 (3.8%)	8/68 (11.8%)	6/64 (9.4%)	
**Total**	**26/158 (16.5%)**	**68/158 (43.0%)**	**64/158 (40.5%)**	
